# Fossils improve extinction-rate estimates under state-dependent diversification models

**DOI:** 10.1098/rstb.2023.0313

**Published:** 2025-02-20

**Authors:** Bruno do Rosario Petrucci, Michael R. May, Tracy A. Heath

**Affiliations:** ^1^ Department of Ecology, Evolutionary, and Organismal Biology, Iowa State University, Ames, IA 50011, USA; ^2^ Department of Evolution and Ecology, University of California Davis, Davis, CA 95616, USA

**Keywords:** state-dependent speciation and extinction, fossilized birth–death process, Bayesian inference, simulation, macroevolution, palaeobiology

## Abstract

The effect of traits on diversification rates is a major topic of study in the fields of evolutionary biology and palaeontology. Many researchers investigating these macroevolutionary questions currently make use of the extensive suite of state-dependent speciation and extinction (SSE) models. These models were developed for, and are almost exclusively used with, phylogenetic trees of extant species. However, analyses considering only extant taxa are limited in their power to estimate extinction rates. Furthermore, SSE models can erroneously detect associations between neutral traits and diversification rates when the true associated trait is not observed. In this study, we examined the impact of including fossil data on the accuracy of parameter estimates under the binary-state speciation and extinction (BiSSE) model. This was achieved by combining SSE models with the fossilized birth–death process. We show that the inclusion of fossils improves the accuracy of extinction-rate estimates for analyses applying the BiSSE model in a Bayesian inference framework, with no negative impact on speciation-rate and state transition-rate estimates when compared with estimates from trees of only extant taxa. However, even with the addition of fossil data, analyses under the BiSSE model continued to incorrectly identify correlations between diversification rates and neutral traits.

This article is part of the theme issue ‘“A mathematical theory of evolution”: phylogenetic models dating back 100 years’.

## 1. Introduction

Questions regarding the tempo and mode of species diversification have always been at the centre of macroevolutionary biology. Yule [1] laid down the groundwork for a century of research that has expanded the mathematical models and statistical methods enabling researchers to explore a vast array of species-diversification patterns in the tree of life. In this foundational paper, Yule [1] introduced a model for the process of lineage accumulation where the per-lineage birth-rate parameter (typically denoted by λ) controls the rate at which new species are generated. Subsequently, while studying Lotka–Volterra processes, Feller [2] extended this stochastic model by introducing a per-lineage death-rate parameter (*μ*) to accommodate species extinction, which was absent in the Yule model. This birth–death (BD) model was later expanded to include time heterogeneity [3], and introduced into major use in the study of palaeobiology [4], macroevolution [5–7] and epidemiology [8,9]. Now an essential tool in statistical phylogenetic analyses, the BD model family has seen continued elaboration to match known biological assumptions, such as incomplete taxon sampling [10,11], density-dependent diversification [12], age-dependent speciation-rate variation [13], serial sampling [14] and much more.

Neontological studies investigating patterns of speciation and extinction typically apply BD models to phylogenetic trees comprising only extant taxa. However, a series of studies has shown that restricting analyses to only extant species leads to difficulties in estimating extinction rates [15,16]. BD models that accommodate non-contemporaneous samples [14] enable the use of fossil occurrences, which have been shown to improve the accuracy of extinction-rate estimates [17,18]. The fossilized BD (FBD) model enables the integration of historical samples by introducing the per-lineage fossil-sampling rate parameter, typically denoted as ψ [14,19,20]. Using simulated datasets under a range of fossil-sampling scenarios, Warnock *et al*. [21] showed that rate-parameter estimates under the FBD model are more accurate, on average, than those estimated using a BD model assuming complete species sampling. In another study, Mitchell *et al*. [22] extended the model of Rabosky [23] to include fossil samples in a branch-wise heterogeneous BD process. They demonstrated that extinction-rate estimates were more accurate with the inclusion of fossil occurrences, including in cases where rates change throughout the history of the tree. Furthermore, both studies showed that parameter estimation under the FBD model is robust to a number of model violations, such as underestimating the number of fossil occurrences. Armed with FBD models (and other non-FBD methods that include fossils in BD models, e.g. PyRate [24]), researchers can now independently estimate speciation, extinction and fossil sampling. Modelling all three parameters is paramount for a full understanding of the diversification history of a group, as their complex interactions can lead to confounding patterns if only a subset is studied.

While the possibility of modelling diversification was innovative, the models discussed above do little to address the causes of such diversification. Many researchers are interested in investigating how diversification rates might be connected to abiotic factors (e.g. related to the environment [25]), whereas others might want to test hypotheses concerning the effect of biotic variables, such as the phenotypic traits of the species in their group of interest. Non-parametric methods like sister-clade analysis (e.g. [26]) can shed some light on these patterns, but such methods are unable to disentangle trait effects on speciation and extinction [27]. Methods and models for understanding trait evolution on a tree, such as independent contrasts [28] or discrete-state character change [29,30], are useful for identifying correlations in trait evolution and other factors. However, because the evolution of traits is considered independently from the lineage-generation process under these approaches, they cannot account for the possibility that a trait of interest may influence diversification, and thus may not adequately model the underlying evolutionary process [31]. To account for trait and diversification-rate associations, Maddison *et al*. [32] introduced the binary state-dependent speciation and extinction (BiSSE) model. BiSSE extends BD models to include two rate categories that evolve following a continuous-time Markov chain, with speciation and extinction rates that vary depending on which state {0,1} a species is in at a given time, i.e. $\left\{\lambda_{0},\lambda_{1}\right\}$ and $\left\{\mu_{0},\mu_{1}\right\}$. This binary-state model was later extended to account for traits with more than two states (multistate speciation and extinction model (MuSSE) [33]), unobserved traits (hidden states speciation and extinction model (HiSSE) [34]), continuous traits (quantitative state speciation and extinction model (QuaSSE) [35]), and many other complex processes. The SSE class of models is now widely adopted (though note that there are non-SSE alternatives to trait-dependent diversification estimation, including with fossils, e.g. [36,37]), and has been used to investigate associations among traits and diversification rates along many of the branches in the tree of life, ranging from nightshades [38], to urban birds [39], to Agaricomycetes fungi [40].

While powerful at detecting state-dependent heterogeneity of speciation rates, SSE models suffer from some distinct limitations. Since the model’s original publication, BiSSE has been known to have low power to detect trait-dependent heterogeneity in extinction rates [32,41], and its extensions fared similarly [33–35]. As discussed above, any BD model that only considers extant data is vulnerable to this problem. SSE models might be even more affected, however, since trait history is an extra source of variation that can bias rates if incorrectly estimated. Furthermore, Maddison & FitzJohn [42] pointed to an issue that is pervasive in methods attempting to detect trait-dependent diversification: within-clade pseudoreplication. If a trait is unique to a few clades, state-dependent diversification analyses might detect spurious trait–rate relationships simply because all species within those clades are pseudoreplicates. While not a focus of this article, this is a limitation to keep in mind when using SSE models, since BiSSE [32] and QuaSSE [35] have also been shown to exhibit that issue.

Rabosky & Goldberg [43] identified another—arguably much more serious—issue in the use of SSE models: their tendency to detect spurious correlations between diversification rates and neutral traits. Since diversification rates are known to vary throughout the tree of life, analyses applying the BiSSE model can erroneously detect a neutrally evolving trait as the source of variation, if it is the only trait available to the model. This means that any such positive result linking a trait to a group’s diversification rates may be spurious, and calls into question the usefulness of these models. A series of subsequent papers offered some solutions to these issues. Rabosky & Goldberg [44] introduced FiSSE, a non-parametric approach that tests whether the trait in question is at all correlated with diversification before the researcher attempts to apply any state-dependent diversification model. Beaulieu & O'Meara [34] showed that hypothesis testing comparing the fit of an SSE model to a model with trait-independent heterogeneous diversification rates, as opposed to a constant-rate model, can reduce the tendency of the model to detect spurious trait-diversification relationships. Most recently, the method by Schwery *et al*. [45] generates posterior predictive distributions for test statistics to evaluate model adequacy. The set of potential limitations associated with SSE models presents a challenge for empirical researchers designing studies and testing hypotheses concerning trait-associated diversification. These tools and exemplary studies—like the work of Zenil-Ferguson *et al*. [38] that used multiple models and hypothesis testing to find the most likely trait–rate associations—continue to provide guidelines for scientists applying SSE models to answer macroevolutionary questions.

A recent study by Beaulieu & O'Meara [46] was the first to evaluate the impact of fossil data on diversification-rate estimates under SSE models, specifically MiSSE (missing-state speciation and extinction [47]), which is a HiSSE variant with only hidden states. One major conclusion of this study was that excluding sampled ancestors (i.e. fossil samples that have sampled descendants) from a dataset can bias estimates of diversification rates. Nevertheless, in contrast to previous simulation studies on trait-independent FBD models [21,22], Beaulieu & O'Meara [46] found that even when all (or a large proportion of) fossils were included, analyses resulted in only minor precision improvements when comparing estimates using trees with only extant taxa to those applied to trees of extant and fossil samples. While the paper made important progress in our understanding of SSE models, many questions remain. Since the authors parametrized their model to estimate turnover (λ+μ) and extinction fraction (μ/λ), as opposed to speciation and extinction, the impact of fossils on the estimates for these untransformed parameters remains unclear. Furthermore, MiSSE differs from other SSE models in the important aspect that it does not include trait data. The inclusion of fossils with trait data might be particularly impactful on other SSE models because of the extra information on the evolution of the trait of focus. It is therefore important to evaluate the performance of state-dependent models when applied to datasets with fossils.

In this study, we used simulated data to examine the impact of including data from fossil taxa on the accuracy of parameter estimates (speciation, extinction and state-transition rates) under the BiSSE model and quantify the error rate in determining associations between traits and diversification rates. A generic state-dependent speciation and extinction model that can accommodate fossil samples is currently implemented in RevBayes [48] with the TensorPhylo plugin [49], which essentially integrates the HiSSE model and the FBD process (for a mathematical derivation, see [46]). We applied RevBayes to simulated datasets generated under a range of scenarios that varied the associations between rates and traits. Our results indicate that the accuracy of extinction-rate estimates is greater when fossil data are included in BiSSE analyses. However, historical observations do not eliminate the tendency for these models to identify spurious associations between neutral trait states and speciation rates. These findings provide a more detailed picture of the limitations of SSE models and will help guide researchers in determining how best to apply macroevolutionary analyses to test hypotheses concerning traits and diversification rates.

## 2. Methods

To gauge whether the addition of fossil samples can improve extinction-rate estimates, we simulated phylogenetic trees, fossil records and trait data. These data were analysed using RevBayes [48], and we compared the results of applying BiSSE to trees with and without fossil samples. The simulated data were generated under different scenarios that varied the trait and rate associations. This allowed us to additionally examine the tendency of the model to falsely detect state-dependent rate heterogeneity in the presence of neutral traits.

### (a) Data generation

We used the R [50] package paleobuddy (v. 1.1.0 [51]) to simulate phylogenetic, fossil and trait data. In our simulations, species evolve according to a BiSSE model, with a binary trait that transitions from state 0 to state 1, and vice versa, with rates $q_{01}$ and $q_{10}$, respectively. Species with trait value i have speciation rate $\lambda_{i}$ and extinction rate $\mu_{i}$. For any given branch in the tree, the trait value at the start of the lineage is equal to the trait value of the parent lineage at the time of speciation, except for the first species which starts in state 0. More traits can evolve in conjunction with species diversification (see description of simulation scenarios below), but only one affects rates. Simulations were run until reaching 100 extant species (discarding replicates that went extinct before reaching that point), following the procedure in Stadler [52]. Fossil occurrences were generated on each tree according to a Poisson process with rate ψ. Note that there is evidence in the fossil record for both time- and lineage-dependence in fossil sampling [53]. While not tested in this article so as to better focus our analyses on speciation and extinction patterns, future work could expand on our analyses by examining the impact of heterogeneous sampling. Each fossil specimen was assigned a trait value corresponding to the state of the species in question at the time of sampling. For each simulation replicate, this procedure yielded a complete tree with extant and extinct species, as well as fossil specimens. We performed rejection sampling to ensure that each trait was present in at least 10% of taxa in both the extant and FBD trees.

In order to evaluate the impact of including fossil occurrences, we modified each complete phylogeny to produce (i) an ultrametric phylogeny containing only extant terminal taxa and (ii) a tree with fossil and extant samples. In all of these, all extant species were included (i.e. extant sampling ρ=1). While this is an unrealistic assumption, it allows us to better isolate the question of whether the addition of fossils improves estimates. The ultrametric trees were generated using the ape package [54] on the complete tree by pruning off extinct lineages. To obtain a tree containing fossil data, we first removed all extinct species that were not sampled in the fossil sampling simulation. Finally, we removed the edges between the last fossil sample of a species and its extinction time, leaving the last fossil sample as a fossil tip and all others as sampled ancestors. For brevity, we refer to these two types of input trees as extant trees and FBD trees, respectively.

**Figure 1.**
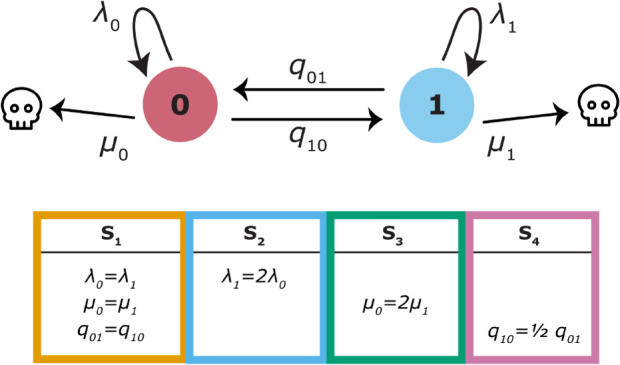
The four simulation scenarios. For each scenario, we ran 100 simulations, each ran until there were 100 extant tips, and then extracted both extant and FBD trees. Speciation and extinction rates for state i are $\lambda_{i}$ and $\mu_{i}$, respectively, and $q_{ij}$ is the transition rate from state i to j. Values used were $\lambda_{0}=0.1$, $\mu_{1}=0.03$ and $q_{01}=0.01$.

We designed a set of four simulation scenarios (summarized in figure 1) to understand the impact of including fossil specimens on rate-estimation accuracy. For each set of simulation conditions, we generated 100 replicate trees and datasets. The constant-rate model, $S_{1}$, is a control case, where rates do not vary with the trait, and transition rates are equal. In $S_{1}$ and all subsequent scenarios, $\lambda_{0}=0.1$, $\mu_{1}=0.03$ and $q_{01}=0.01$. In $S_{2}$, the speciation rate associated with state 1 is double that of state 0, i.e. $\lambda_{1}=2\lambda_{0}$. In $S_{3}$, the extinction rate associated with state 0 is double that of state 1, i.e. $\mu_{0}=2\mu_{1}$, while the speciation rate remains the same for both states (note that the diversification rate is higher for state 1 in both cases). Finally, in $S_{4}$, the transition rate from state 1 to state 0 is half of the transition rate from 0 to 1, i.e. $q_{10}=\frac{1}{2}q_{01}$, and speciation and extinction rates do not vary. Note that these are the same simulation scenarios used to test BiSSE in Maddison *et al*. [32], so as to make our results comparable with those tests. For each set of simulations, we also generated neutral traits evolving alongside the focal trait, but these traits were not associated with diversification rates. Partially following Rabosky & Goldberg [43], we simulated slow-, medium- and fast-evolving neutral traits, with symmetrical transition rates equal to 0.01, 0.1 and 1, respectively. Finally, we simulated fossil sampling under three different rates, ψ=0.01, 0.05 and 0.1. As with the true diversification-influencing trait, we ensured each neutral trait had at least 10% representation of each state using rejection sampling.

With our four simulation scenarios, four fossil sampling rates (including ψ=0 for an extant tree) and 100 replicates per configuration, we generated 1600 trees. For the average number of species (extant and extinct), average simulation duration and average number of fossils sampled for each combination of simulation parameters, see electronic supplementary material, table S1. Four sets of traits were generated on each of the trees, leading to a total of 6400 tree and trait datasets. For access to the simulation and analysis files, please see the details in the Data accessibility section.

### (b) Analyses

To analyse our simulated datasets under the BiSSE and BiSSE+FBD models, we used RevBayes [48] with the TensorPhylo plugin [49], which implements SSE models already present in RevBayes with greater computational efficiency. We specified lognormal priors on $\lambda_{i}$, $\mu_{i}$ and $\psi_{i}$ (when applicable). While the model allows for trait-dependent fossil sampling as well as diversification rates, that is not a pattern examined in this study. For the transition-rate parameter, we used an exponential with a mean equal to the transition rate value used for the focal trait in each simulation, 0.01. We fixed the sampling probability at present to ρ=1 as we included all extant species for all analyses. Note that we specified priors for our RevBayes analyses such that the true parameters used to generate the data had non-zero probability. In this way, we avoid confounding noise due to prior misspecification when comparing BiSSE and BiSSE+FBD. Future work could investigate how robust these results are to violations of model assumptions. We ran each analysis for 1 00 000 Markov chain Monte Carlo (MCMC) iterations, with 29 moves per iteration, tuning every 200. The analyses were all run in the Iowa State University high-performance computing clusters Nova and Pronto. Convergence was assessed by calculating ESS values using the package coda [55] and ensuring ESS values for all parameters were above 200. Hereafter, we refer to each group of 100 analyses run with identical RevBayes and paleobuddy settings as an analysis set.

Each analysis in RevBayes logs the MCMC samples for every parameter, and we summarized the posterior distributions for $\lambda_{i}$, $\mu_{i}$ and $q_{ij}$. For each analysis set and parameter, we calculated 95% coverage proportions (the proportion of analyses where the true value is contained in the 95% credible interval), which should be close to 0.95 if the model is behaving as expected [56]. Note, however, that since we did not draw simulation parameter values from the priors but instead used a set of fixed values, we do not necessarily expect 0.95 coverage for all simulation settings. Then, we calculated the posterior means of $\lambda_{i}$, $\mu_{i}$ and $q_{ij}$ for each analysis in the set, and summarized these values to visualize and compare estimates. Finally, we used these means to calculate relative errors, such that (using $\lambda_{i}$ as an example):


$$\begin{array}{r} \text{error}=\frac{|\bar{\lambda_{i}}-\lambda_{i}|}{\lambda_{i}}, \end{array}$$

where the absolute difference between the posterior mean rate estimate $\bar{\lambda_{i}}$ and the true speciation rate $\lambda_{i}$ for a given state i is divided by the true rate. We used these summary statistics to visualize and quantify accuracy and compare the impact of including fossil data.

To visualize the tendency of BiSSE to detect spurious trait–diversification interactions, we calculated the posterior probability of $\lambda_{1}>\lambda_{0}$ for each analysis set, including those inferred using a neutrally evolving trait. This is similar to the procedure in Rabosky & Goldberg [43], except for our use of a Bayesian posterior probability, instead of a frequentist *p*‐value.

## 3. Results

### (a) Fossil specimens improve extinction-rate estimates

**Figure 2.**
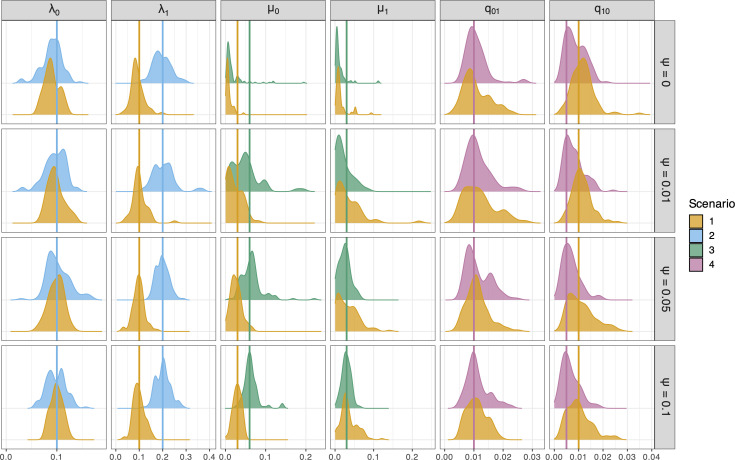
Density ridge plot for the mean of the posterior distribution of each rate of focus in scenarios 1–4. Scenario number and colour refer to the conditions laid out in figure 1. Speciation and extinction rates for state i are $\lambda_{i}$ and $\mu_{i}$, respectively, and $q_{ij}$ is the transition rate from state i to j. Vertical lines show the true simulated value of each rate. Analyses of only extant taxa are labelled with ψ=0, while other rows show analyses of trees with fossil records simulated with increasing fossil-sampling rate. For density plots of all rates for each scenario, see electronic supplementary material, figure S3.

To compare the parameter estimates from BiSSE and BiSSE+FBD, we plotted the distributions of posterior means for all replicates in each simulation scenario in figure 2, using only the focus, i.e. non-neutral, trait. We did not include ψ estimates in this figure as this parameter was fixed in all simulations (i.e. $\psi_{0}=\psi_{1}$). Note, however, that BiSSE+FBD was able to estimate the true value of $\psi_{0}$ and $\psi_{1}$ with reasonable accuracy (see electronic supplementary material, figures S1 and S2). First, we will focus on the top row of figure 2, which shows BiSSE estimates from extant data only (ψ=0), and reveals a pattern familiar from Maddison *et al*. [32]. Speciation-rate estimates are shown in the first two plots ($\lambda_{0}$ and $\lambda_{1}$ with ψ=0 in figure 2), and our results indicate that heterogeneity is correctly detected, with the distributions of $\lambda_{0}$ and $\lambda_{1}$ posterior means centred on the true values when rates are equal ($S_{1}$) and when speciation rate is correlated with the trait ($S_{2}$). Our results also show that estimates of transition rates $q_{01}$ and $q_{10}$ are accurate, on average; however, there is variation across the replicate simulations ($q_{01}$ and $q_{10}$ with ψ=0 in figure 2). Estimates of extinction rates from datasets comprising only extant taxa are less accurate ($\mu_{0}$ and $\mu_{1}$ with ψ=0 in figure 2), and the posterior means are concentrated at values very close to 0, matching the known behaviour of these models [16].

When fossil specimens are included in the data and model (ψ=0.01, 0.05 or 0.1 shown in the bottom three rows of figure 2), we continue to see accurate estimates of speciation rates ($\lambda_{0}$, $\lambda_{1}$) and state-transition rates ($q_{01}$, $q_{10}$), matching the performance of the analyses of only extant taxa described above. Extinction-rate estimates, however, show increasing accuracy with increasing values of ψ. The distributions of posterior-mean estimates shown in the centre columns of figure 2 ($\mu_{0}$ and $\mu_{1}$ with ψ=0.01, 0.05 or 0.1) are centred on the true values even with very low fossil sampling rates (ψ=0.01). The last row shows that when ψ=0.1, the heterogeneity in extinction rates is recovered for a large percentage of simulations, with narrow overlap between posterior-mean distributions of $\mu_{0}$ estimates for $S_{1}$ and $S_{3}$. Note that to better focus on the patterns of interest, figure 2 plots only the rates that vary in scenarios $S_{2}$ through $S_{4}$. For a complete plot of all rates, see electronic supplementary material, figure S3.

**Figure 3.**
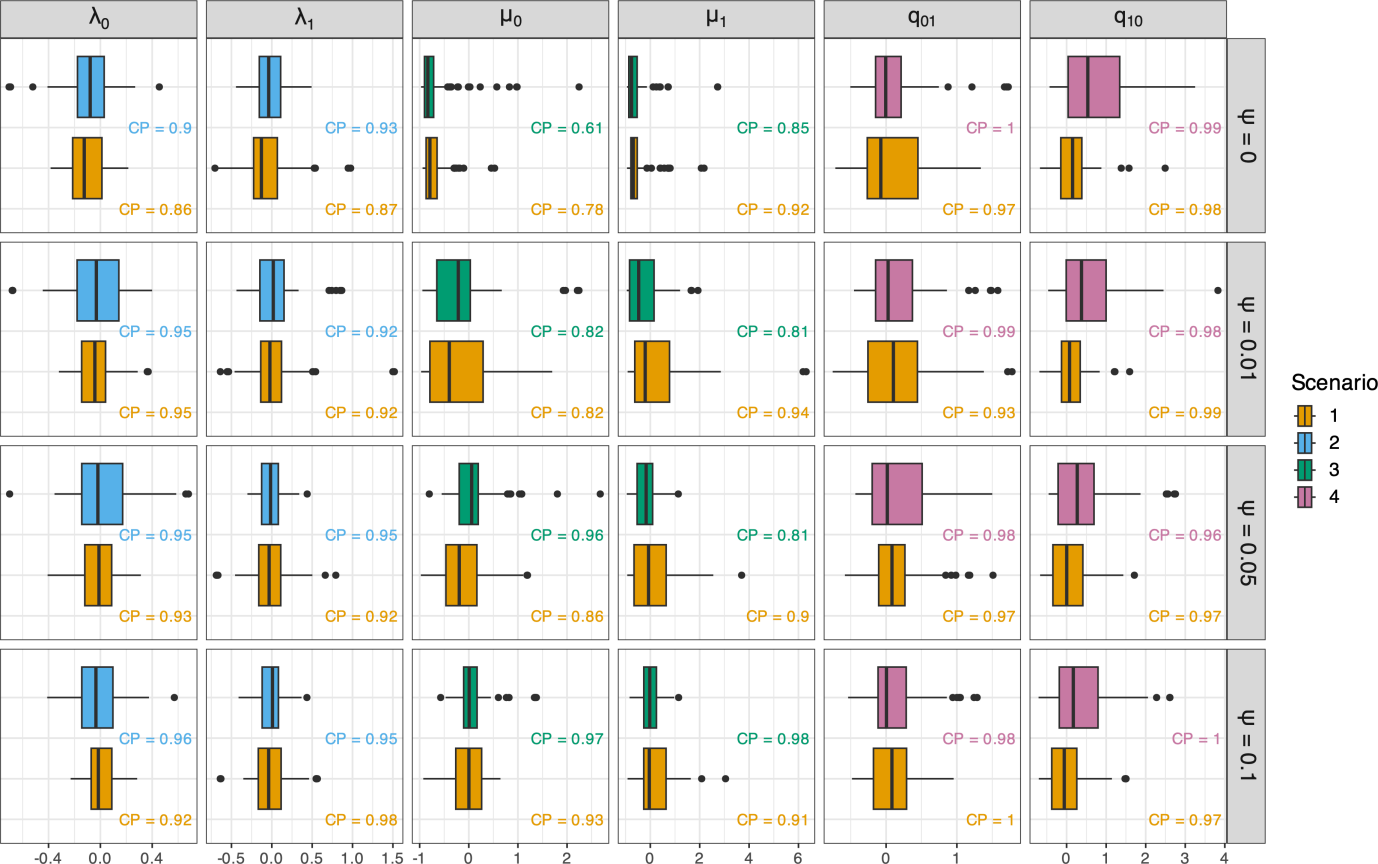
Boxplots of relative error for mean estimates of each rate of focus in scenarios 1−4. Scenario number and colour refer to the conditions laid out in figure 1. Speciation and extinction rates for state i are $\lambda_{i}$ and $\mu_{i}$, respectively, and $q_{ij}$ is the transition rate from state i to j. Coverage proportion (CP) values for each scenario are written underneath the corresponding boxplot. Analyses of trees with only extant taxa are labelled with ψ=0, while all other rows show analyses of trees with fossil records simulated with increasing fossil-sampling rate.

To further summarize these results, we calculated relative error for each rate across all analyses, and the 95% coverage proportion (the proportion of replicates where the true value of the rate was in the 95% confidence interval of the posterior distribution) for each simulation scenario (summarized in figure 3). These results support the interpretation based on figure 2 above: speciation rates and state-transition rates are estimated with relatively high accuracy both with and without fossils, but fossil data improve extinction-rate estimates. The relative error is concentrated at 0 and coverage proportion is close to 0.95 for all estimates of speciation rate ($\lambda_{0}$, $\lambda_{1}$) and transition rates ($q_{01}$, $q_{10}$). When fossil data are ignored (ψ=0), however, extinction rates ($\mu_{0}$, $\mu_{1}$) are almost always underestimated, with an excess of negative values for relative error and lower coverage proportions (figure 3). These results indicate that fossils can increase the accuracy of extinction-rate estimates without affecting speciation- and transition-rate estimates.

### (b) Datasets with fossils detect spurious trait and diversification-rate associations

**Figure 4.**
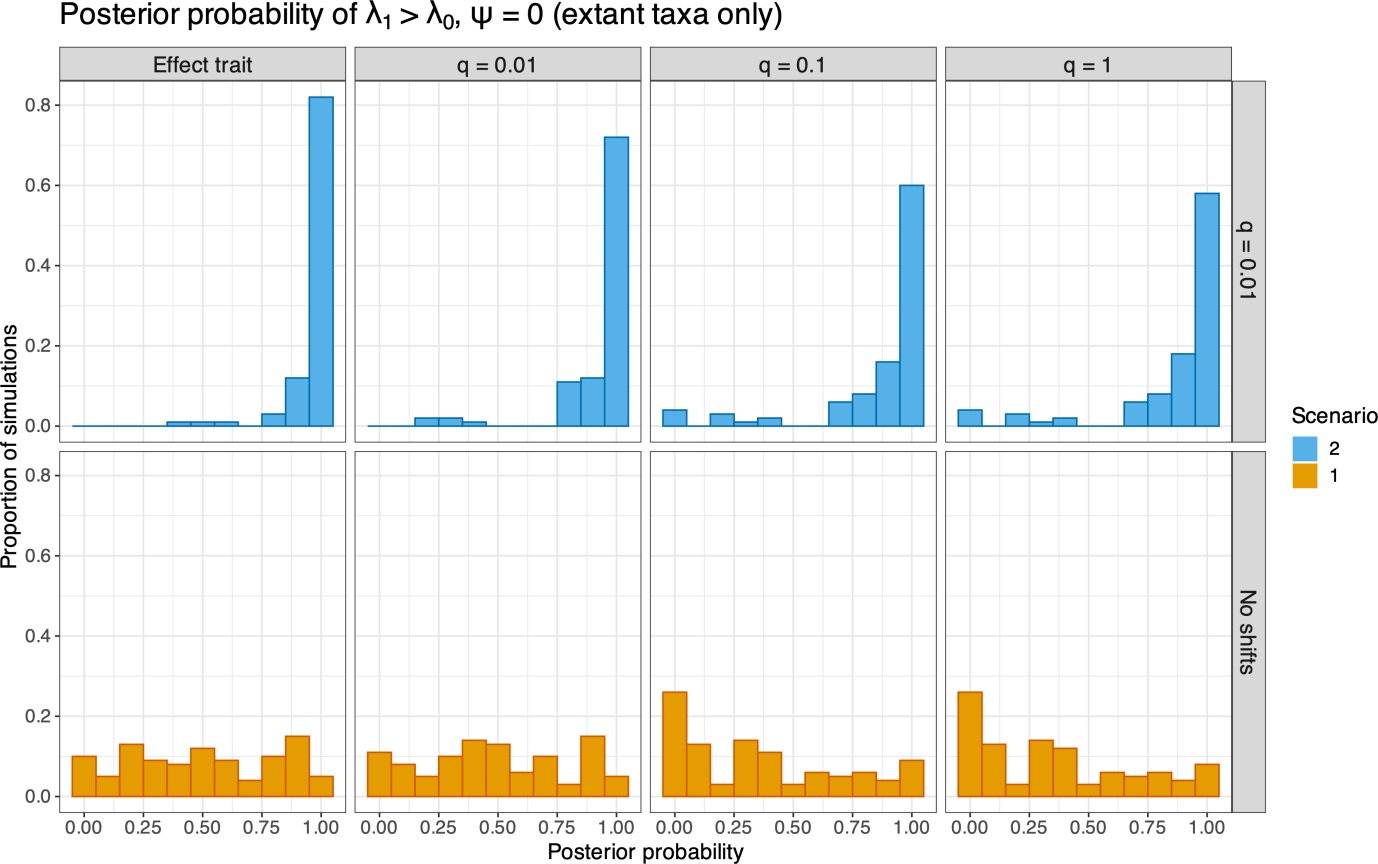
Histograms of the posterior probability of $\lambda_{1}>\lambda_{0}$ (the speciation rates for state 1 and 0, respectively) for analyses of 100 simulated datasets comprising only extant taxa generated under $S_{1}$ (bottom row) and $S_{2}$ (top row). The effect trait is shown in the first column, and neutral traits with increasing symmetrical transition rates (q=0.01, 0.1 and 1) are shown for the subsequent columns. Scenario 1 ($S_{1}$) refers to the case where $\lambda_{0}=\lambda_{1}$, and scenario 2 ($S_{2}$) where $\lambda_{1}=2\lambda_{0}$, for the effect trait.

**Figure 5.**
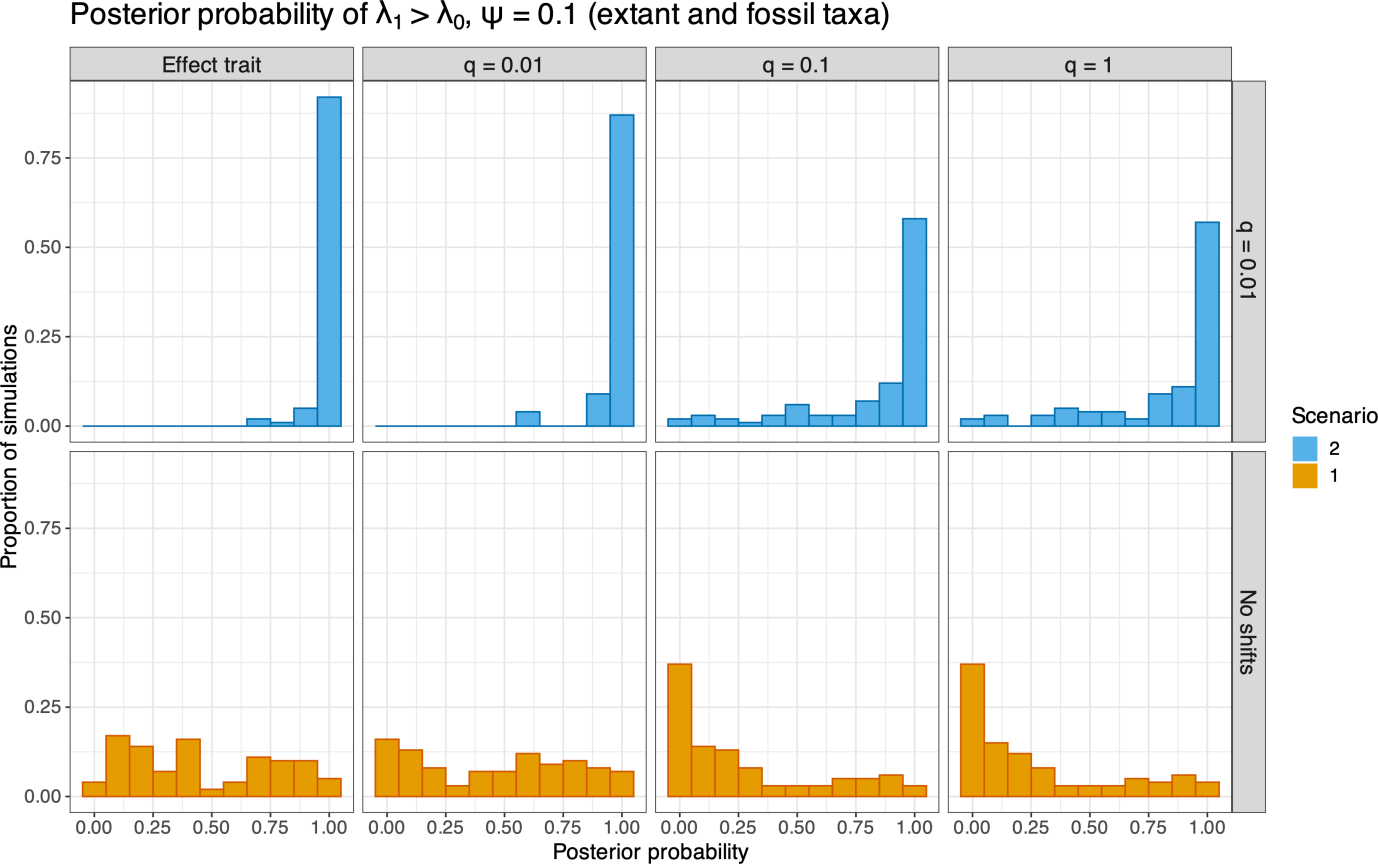
Histograms of the posterior probability of $\lambda_{1}>\lambda_{0}$ (the speciation rates for state 1 and 0, respectively) for analyses of 100 simulated datasets comprising both extant and fossil taxa generated under $S_{1}$ (bottom row) and $S_{2}$ (top row). The effect trait is shown in the first column, and neutral traits with increasing symmetrical transition rates (q=0.01, 0.1 and 1) are shown for the subsequent columns. Scenario 1 ($S_{1}$) refers to the case where $\lambda_{0}=\lambda_{1}$, and scenario 2 ($S_{2}$) where $\lambda_{1}=2\lambda_{0}$, for the effect trait.

State-dependent speciation and extinction models have been shown to erroneously identify correlations between neutral traits and diversification rates [43]. To evaluate the propensity of falsely detecting trait–rate associations under the BiSSE model, we calculated the posterior probability of $\lambda_{1}>\lambda_{0}$ estimated for each replicate in scenario $S_{1}$, which constrained $\lambda_{1}=\lambda_{0}$, as well as for replicates in scenario $S_{2}$, which generated data under the condition $\lambda_{1}=2\lambda_{0}$. We computed the posterior probabilities of $\lambda_{1}>\lambda_{0}$ for both the effect trait (i.e. the trait correlated with diversification) and neutral traits. These results are presented in a set of histograms shown in figure 4 for extant-only datasets and figure 5 for analyses that included fossil samples (these histograms are similar to fig. 4 of [43]). Values closer to 1 indicate that the model detected trait-dependent heterogeneity in a higher proportion of datasets. Figure 4 shows the results for analyses of trees comprising only extant taxa (ψ=0). For analyses of data simulated under scenario $S_{1}$ (figure 4, bottom row), we see that $P(\lambda_{1}>\lambda_{0})$ has a uniform distribution, which is expected since the true model constrained $\lambda_{0}=\lambda_{1}$. Under the $S_{2}$ generating model that specified trait-dependent speciation rates (figure 4, top row), the effect trait column shows that $P(\lambda_{1}>\lambda_{0})$ is close to 1 across our simulation replicates. This is also expected under the true model, where $\lambda_{1}=2\lambda_{0}$. We show histograms for $P(\lambda_{1}>\lambda_{0})$ for unassociated traits with increasing symmetrical transition rates (q=0.01, 0.1 and 1) in columns 2−4 of figure 4. These results show that $P(\lambda_{1}>\lambda_{0})$ is also very high when only neutral traits are considered along with trees generated under scenario $S_{2}$. This is consistent with the behaviour observed by Rabosky & Goldberg [43], confirming that analyses under the BiSSE model are prone to assigning heterogeneous diversification–rate associations to neutral traits when the effect trait is not observed. We present the summary of $P(\lambda_{1}>\lambda_{0})$ for analyses of extant and fossil (ψ=0.1) data generated under scenarios $S_{1}$ and $S_{2}$ in figure 5. The same was observed for lower values of ψ (see electronic supplementary material, figures S4 and S5). The pattern of spurious neutral-trait and speciation-rate associations remains, even with the inclusion of fossil specimens.

The bottom row of figure 5 (and, to a lesser extent, figure 4) shows a somewhat unexpected pattern for neutral traits when rates are equal ($S_{1}$), where the posterior probabilities of $\lambda_{1}>\lambda_{0}$ seem to concentrate at or near 0, instead of being more uniformly distributed. While our trait simulations all started with a root state of 0, our analyses estimated the parameter $\pi_{0}$ (the probability of the root state starting at 0) instead of fixing it to 1, which is common practice in applications of the BiSSE model. Thus, for high transition-rate values (q), the analysis was less likely to detect the trait history and a root state starting at 0 (see electronic supplementary material, figure S6). This resulted in a higher estimated ratio of $\lambda_{0}/\lambda_{1}$ to explain the higher prevalence of species with state 0 (due to its identity as the root state). While this pattern does not change our conclusions, it raises interesting questions about the role of root state probability estimates in SSE models, and should be considered for further investigation in future studies.

**Figure 6.**
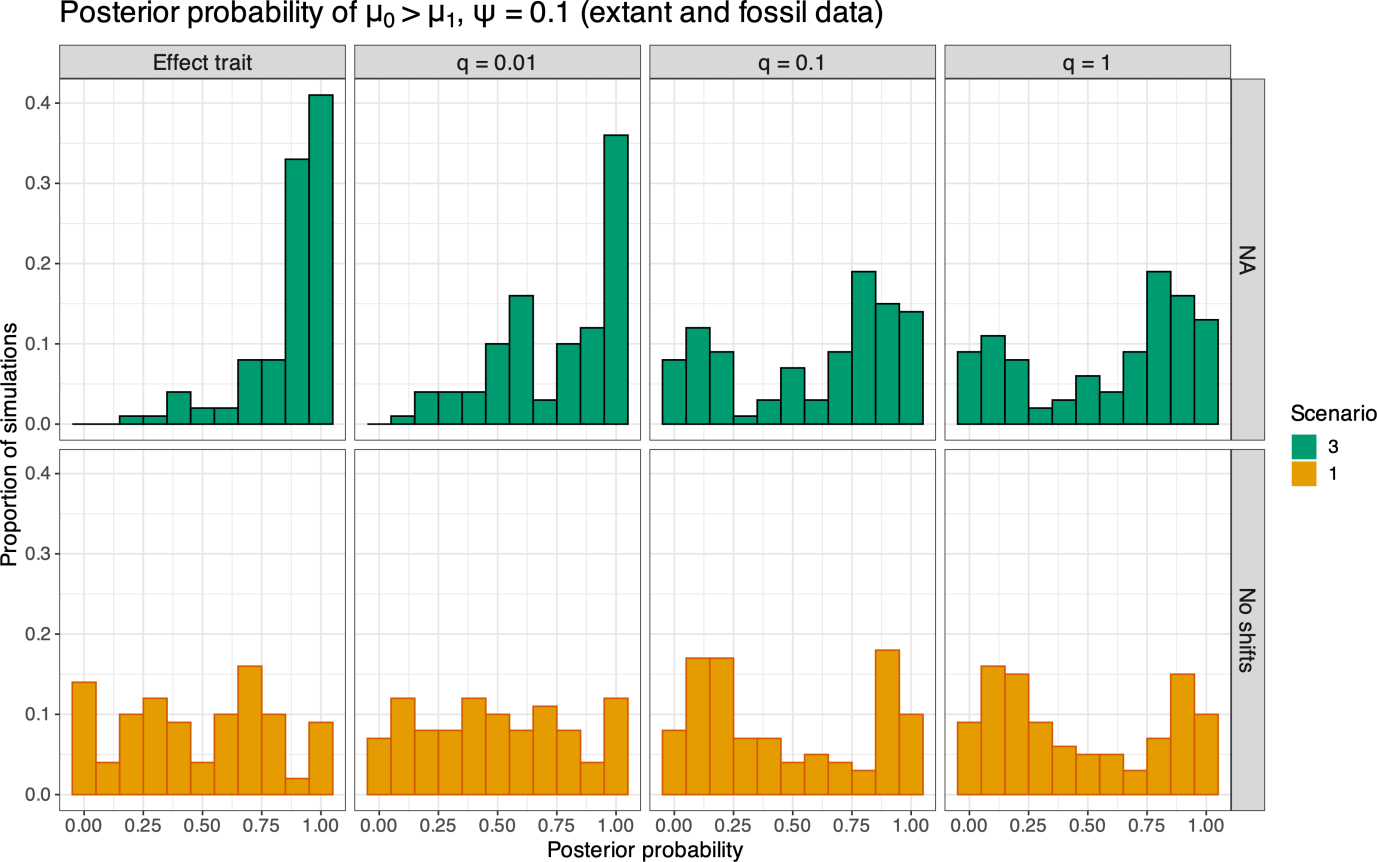
Histograms of the posterior probability of $\mu_{0}>\mu_{1}$ (the extinction rates for state 0 and 1, respectively) for analyses of 100 simulated datasets comprising both extant and fossil taxa (ψ=0.1) generated under $S_{1}$ (bottom row) and $S_{3}$ (top row). The effect trait is shown in the first column, and neutral traits with increasing symmetrical transition rates (q=0.01, 0.1, and 1) are shown for the subsequent columns. Scenario 1 ($S_{1}$) refers to the case where $\mu_{0}=\mu_{1}$, and scenario 3 ($S_{3}$) where $\mu_{0}=2\mu_{1}$, for the effect trait.

Our simulations show that fossil data help inform extinction rate estimates, yet analyses of these data under the BiSSE model can also result in spurious associations between traits and extinction rates. Figure 6 shows the posterior probability of $\mu_{0}>\mu_{1}$ for $S_{3}$ (where $\mu_{0}=2\mu_{1}$). Similar to estimates when speciation rates vary (as shown in figure 5), we see a higher proportion of simulation replicates with posterior probabilities at or near 1 for $\mu_{0}>\mu_{1}$ when the trait is truly neutral (top row of figure 6), though this pattern is less severe when extinction rates are trait-dependent. This is likely due to the reduced accuracy of extinction-rate estimates, as shown by the higher per cent error for many simulation replicates in figure 3. Electronic supplementary material, figures S7 and S8 also show the posterior probability of $\mu_{0}>\mu_{1}$ for $S_{3}$, with decreasing values of fossil sampling ψ (0.01 and 0.05, respectively). These results show that, as the rate of fossil sampling (ψ) increased, a larger proportion of simulation replicates detected extinction-rate associations with neutral traits (with posterior probability equal to 1). Electronic supplementary material, figure S9 shows the receiver operating characteristic (ROC) curve for all the trait–rate association tests, where one can visualize the expected false-positive rate for a given true-positive rate.

## 4. Discussion

A century ago, the innovative model described by Yule [1], enabled evolutionary biologists to answer questions about lineage diversification. The sole birth parameter, however, could not fully describe the complex processes responsible for generating observed biodiversity. The fossil record and the age of life on Earth indicate that the vast majority of lineages in the tree of life have gone extinct [57]. Thus, incorporating extinction rates into a model of species diversification is necessary for accurately investigating these patterns. For example, modelling extinction allows researchers to investigate whether mass-extinction events are due to high extinction rates or low speciation rates [58], or whether dinosaurs were experiencing declining speciation rates before the K–Pg extinction event [59,60]. Accommodating extinction in the BD model [2,3] was a critical development that further elucidated the dynamics of macroevolutionary processes. However, models and methods applied to only extant diversity [5,6] can be limited in their ability to accurately estimate extinction rates [15]. Fossil samples allow researchers to observe ancient biodiversity, and the inherent rarity of fossilization means accurate modelling of fossil sampling is also critical for statistical methods seeking to illuminate species diversification.

The FBD process established a coherent framework for integrating both palaeontological and neontological data in diversification-rate analyses [14,19,20]. Analyses under the FBD model allow researchers to make better use of data from the fossil record, especially for fossil-rich clades. These studies have resulted in divergence-time estimates that are more consistent with the fossil record (e.g. [61]), helped resolve difficult phylogenetic relationships (e.g. [62,63]) and improved our understanding of ancient diversification patterns (e.g. [60]). Moreover, dated phylogenies estimated under the FBD model are frequently used in downstream analyses to test hypotheses about biogeography (e.g. [64]) and the evolution of phenotypic traits (e.g. [65]). Making full use of available paleontological data is particularly important when considering morphological traits, as fossils have also been shown to improve ancestral-state estimates of both continuous [66] and discrete [67] characters. The possibility of looking into the past not only for data related to the timing of diversification but also the evolution of the traits of interest themselves, makes fossil information even more important for questions regarding the effect of traits on diversification. All of this together means that understanding the impact of adding fossils to state-dependent diversification analyses is imperative for researchers interested in studying these patterns.

The results of our simulation study demonstrate that the analysis of both fossil and extant taxa under the BiSSE model yields more accurate estimates of extinction rates, as well as preserving the accuracy of speciation and transition rates, when compared with analyses considering only extant data. It is important, however, that models used to describe state-dependent speciation and extinction allow for sampled ancestors. Previous work has demonstrated that data generated under a FBD model with ψ>0 will have a non-zero probability of including fossil samples with sampled descendants, i.e. sampled ancestors [14,68–70]. Therefore, not only is it important to include information from the fossil record, but our diversification models and methods must also treat these data appropriately to avoid potential bias in our estimates [46]. Furthermore, our results show that extinction-rate accuracy increases with increasing rates of fossil recovery; thus, analyses applied to clades with rich fossil records are likely to produce more accurate extinction-rate estimates than those considering groups with very few fossils. These limitations are unavoidable, however, as the quality of the data’s representation of past dynamics is always likely to have an impact on the quality of estimates. Finally, we point out that the analyses in this simulation study were carried out with fixed phylogenies, so as to better emulate empirical studies using SSE models, and did not therefore consider uncertainty or error in the tree topology or divergence times as possible sources of bias. The inclusion of fossil data in phylogenetic analyses can often lead to greater uncertainty, which may then lead to greater uncertainty in downstream estimates, ultimately making the improvements we observe in extinction-rate estimates less noticeable. While a full investigation of these patterns is not within the scope of this study, we believe this is an important consideration researchers must keep in mind when applying these models to empirical data. Our work, along with previous studies investigating the power and performance of state-dependent diversification models [32,34,41,46], indicate that, given data that adequately represent the diversification history of the group in question, analyses under the BiSSE model can produce accurate results about their past dynamics. BiSSE analyses applied to datasets of only extant taxa can detect trait-associated heterogeneity in speciation, but questions regarding trait-dependent extinction are difficult to explore using ultrametric trees. These questions are more relevant than ever as researchers seek to understand and mitigate the current biodiversity crisis [25,71–73], since a better understanding of trait-associated extinction in the past can aid in studying similar patterns unfolding in the present.

Despite the improved accuracy of extinction-rate estimates, including fossil data does not eliminate the BiSSE model’s tendency to detect associations between neutral traits and diversification rates when the effect trait is not considered. Our analyses showed a similar distribution of posterior probabilities supporting incorrect trait-dependent diversification rates for both analyses of extant-only trees and FBD trees. This shows that while fossils can reduce some of the shortcomings associated with the BiSSE model, they are not able to remove all of the limitations. We also note that while BiSSE was the only model evaluated here, one would expect MuSSE [33] to behave similarly given that it is a straightforward extension of BiSSE. The HiSSE model [34], on the other hand, is known to be less prone to identifying spurious trait and diversification-rate relationships, compared with BiSSE and other SSE models. Given that HiSSE is also a straightforward mathematical extension to BiSSE, it is likely that one would see similar improvements in extinction rate accuracy when applying HiSSE to fossil data. As such, running HiSSE and BiSSE models to test various hypotheses about the interactions between the traits and rates of interest (e.g. [38]) may be a preferred workflow to uncover extinction-rate patterns, while accounting for known model limitations.

Our results seemingly conflict with the conclusions of Beaulieu & O'Meara [46], where the authors found that fossils do not improve on the accuracy of rate estimates when compared with analyses of extant trees. This conflict might be due to the different way the authors parameterized the fossil-BiSSE model in their study, however. In their paper, Beaulieu & O'Meara [46] estimated turnover (τ=λ+μ) and extinction fraction (ϵ=μ/λ), instead of estimating λ and μ directly. Consequently, they observed high τ and ϵ accuracy when analysing trees containing only extant tips. To understand this pattern, consider that in Maddison *et al*. [32] (where λ and μ were estimated directly), extinction rate estimates were extremely imprecise rather than inaccurate. While we did not recover the same pattern, this might be due to the differences between Bayesian and maximum-likelihood estimates (both [32] and [46] used maximum-likelihood inference). It is likely that the extinction-rate estimates in Beaulieu & O'Meara [46] were similarly imprecise. Furthermore, SSE models can accurately estimate speciation rates, even when fossils are not included. Thus, the accuracy of τ and ϵ found by Beaulieu & O'Meara [46] is likely controlled by the high accuracy of λ estimates, since μ is imprecise and therefore uninformative. When our results are transformed to show τ and ϵ estimates (see electronic supplementary material, figure S10), we recover a similar pattern to Beaulieu & O'Meara [46], further supporting this rationale. We show that the addition of fossils improves the estimates of μ, but since estimates of μ based on extant-only datasets in Beaulieu & O'Meara [46] may have been imprecise, the impact of adding fossils only manifests as an improvement in precision for τ and ϵ. Therefore, the conclusion that extinction rate estimates become more accurate with the inclusion of fossils does not necessarily conflict with the Beaulieu & O'Meara [46] results.

## 5. Conclusion

As observers of Earth’s biodiversity, our species has always sought to understand the processes that generated the patterns we see in the tree of life. The contributions of Yule [1] and the many other evolutionary, epidemiological and mathematical scientists that followed have helped illuminate the macroevolutionary dynamics of our current biodiversity and its history. This simulation study continues this task. We showed that analyses using state-dependent speciation and extinction models to investigate associations between traits and diversification rates benefit from the inclusion of fossil data, especially when testing hypotheses concerning extinction rates. While some of the BiSSE model’s limitations persist, even with fossil data (i.e. spurious detection of associations between diversification and neutral traits), BiSSE and its extensions (especially those less vulnerable to such limitations) remain powerful tools, and the evidence presented here expands the use cases of these models.

## Data Availability

Simulations in this manuscript were run using the R package paleobuddy v. 1.1.0, present in CRAN and GitHub [74]. Analyses were run in RevBayes [48] using v. 1.2.4. All simulation, analysis and data analysis scripts can be found in GitHub [75]. All of the files necessary to generate simulations and analyses are available in a public repository [76]. Supplementary material is available online [77].
